# Bacterial Profile of Dentine Caries and the Impact of pH on Bacterial Population Diversity

**DOI:** 10.1371/journal.pone.0092940

**Published:** 2014-03-27

**Authors:** Nima Kianoush, Christina J. Adler, Ky-Anh T. Nguyen, Gina V. Browne, Mary Simonian, Neil Hunter

**Affiliations:** 1 Institute of Dental Research, Westmead Centre for Oral Health and Westmead Millennium Institute, Westmead, Sydney, Australia; 2 Department of Oral Biology, Faculty of Dentistry, University of Sydney, Sydney, Australia; University of Florida, United States of America

## Abstract

Dental caries is caused by the release of organic acids from fermentative bacteria, which results in the dissolution of hydroxyapatite matrices of enamel and dentine. While low environmental pH is proposed to cause a shift in the consortium of oral bacteria, favouring the development of caries, the impact of this variable has been overlooked in microbial population studies. This study aimed to detail the zonal composition of the microbiota associated with carious dentine lesions with reference to pH. We used 454 sequencing of the 16S rRNA gene (V3–V4 region) to compare microbial communities in layers ranging in pH from 4.5–7.8 from 25 teeth with advanced dentine caries. Pyrosequencing of the amplicons yielded 449,762 sequences. Nine phyla, 97 genera and 409 species were identified from the quality-filtered, de-noised and chimera-free sequences. Among the microbiota associated with dentinal caries, the most abundant taxa included *Lactobacillus sp.*, *Prevotella sp.*, *Atopobium sp.*, *Olsenella sp.* and *Actinomyces sp.* We found a disparity between microbial communities localised at acidic versus neutral pH strata. Acidic conditions were associated with low diversity microbial populations, with *Lactobacillus* species including *L. fermentum*, *L. rhamnosus* and *L. crispatus*, being prominent. In comparison, the distinctive species of a more diverse flora associated with neutral pH regions of carious lesions included *Alloprevotella tanerrae*, *Leptothrix* sp., *Sphingomonas* sp. and *Streptococcus anginosus*. While certain bacteria were affected by the pH gradient, we also found that ∼60% of the taxa associated with caries were present across the investigated pH range, representing a substantial core. We demonstrated that some bacterial species implicated in caries progression show selective clustering with respect to pH gradient, providing a basis for specific therapeutic strategies.

## Introduction

Dental caries is the most prevalent chronic disease of mankind [1]. In regard to cost and quality of life, oral diseases including caries, pose a major burden in developed countries and an increasing problem in developing countries. Advances have not yet had a global impact on caries prevention, with current epidemiological studies indicating a marked increase in prevalence of dental decay among all age groups [2]. The rise in prevalence of caries is despite the proven efficacy of fluoride use and application in control of this disease. Children, immigrants and low socio-economic groups are those mainly affected by this trend [3].

Dental caries is caused by acids produced by commensal microbes within oral biofilms known as plaque [4]. Organic acids, including acetic, lactic and propionic, produced as the by-products of fermentation, dissolve the hydroxyapatite component of enamel and dentine [5] leading to tooth surface breakdown and subsequent cavity formation. In the cavitated lesion, exposed dentine collagen fibres are also subject to enzymatic degradation by bacteria [6].

In most classical medical infections, a single pathogen is responsible for the disease and this pathogen may be present in an otherwise sterile site or a site that is not naturally host to the isolated species [7]. In comparison, caries is a polymicrobial infection mediated by commensal oral microbes. The oral environment is home to diverse bacterial populations, with the Human Oral Microbiome Database (HOMD) documenting the presence of approximately 600 prokaryote species [8]. Considering this large diversity of microbiota in the oral ecosystem, our understanding of the microbial aetiology of caries and how environmental conditions in the oral cavity impact the disease process continues to change as technology advances.

Traditional culture and culture-independent techniques have provided low-level resolution information regarding the microbiota associated with caries. Culture studies showed that *Streptococcus mutans* is the chief pathogen associated with caries [9], [10], in addition to *Lactobacillus* spp. and *Actinomyces* spp. [9], [11]. The identification of a small number of bacteria associated with caries led to the proposed ‘specific plaque hypothesis’ [10], [12], [13]. This resulted in chemical and immunological therapeutic approaches being directed against specific microbial targets [14]–[17]. However, caries has been found to occur in the absence of *S. mutans* [17]–[20].

The lack of involvement of *S. mutans* in some lesions led to the proposal for the ‘mixed/non- specific microbial hypothesis’, formulated to include other acid producing bacteria in caries initiation and progression [11], [21]. Culture-independent studies provided support for this hypothesis, revealing a greater diversity of bacteria associated with caries [22]–[24]. Clonal analysis of the 16S rRNA gene revealed that a diverse array of bacteria including *S. mutans*, non-mutans streptococci and members of the genera *Actinomyces*, *Bifidobacterium, Lactobacillus, Propionibacterium*, *Veillonella, Selenomonas and Atopobium* are associated with different stages of carious lesions [20], [22], [25].

Neither the specific or non-specific microbial hypotheses highlight the impact of ecological variables on microbiota associated with caries. To address this, the ‘ecological plaque hypothesis’ was proposed, which suggested the biofilm be considered as a bacterial community and caries as a ‘dysbiosis’ caused by the changing makeup of the oral microbiota in response to changing environmental variables [4], [7], [26]. Fundamental variables suggested to influence the genotypic and phenotypic characteristics of the oral microbiota include: nutrient availability, oxygen concentration and pH [4], [7], [26]. While the former two factors cannot be assessed accurately in a complex polymicrobial community, pH of a carious lesion can be measured with relative accuracy. Low environmental pH was proposed to cause a shift to an acid-tolerant and acid-producing consortium of bacteria, which alters the balance from remineralisation to demineralization, hence favouring the formation of lesions [26].

The capacity of many aciduric and acidogenic bacterial species to lower the pH of the environment has been reported [27]–[29]. However, knowledge of how the oral bacterial community shifts with pH gradient in caries is restricted to chemostat-controlled studies, which are limited to assessing a small number of species [30]. The advent of next-generation sequencing techniques, such as pyrosequencing, enables in-depth analysis of microbial communities. The recent application of pyrosequencing to determine which microbial taxa are associated with dental caries has focused on documenting variation between caries-active and -free bacterial communities in plaque and saliva [31]–[33]. These studies, while documenting the changing community structure between health and caries with in-depth sequencing, do not reveal how key ecological variables, such as pH, influence the oral microbial community structure. Therefore, the *in vivo* relevance of the ecological plaque hypothesis is yet to be confirmed.

We used 454 sequencing technology to examine the bacterial community associated with dentine caries and to demonstrate how the diversity is influenced by the pH of dentinal cavitated lesions. The pH within a lesion was found to strongly affect the microbial composition of 42% of the dentine caries associated bacteria. This information enabled us to propose a ‘substantial’ core model [34] of the bacterial profile of dentine caries with reference to pH gradient.

## Materials and Methods

### Ethics statement

The NSW Health Ethics committee approved the study design, sampling method and written consent forms (Protocol Number X07-0261). Verbal and written consents were obtained from all participants in this study.

### Sampling

Teeth with occlusal or proximal caries (n = 25) that had open cavities and a diagnosis of irreversible pulpitis were extracted with verbal and written informed consent from adult patients of European, Asian or African descent residing in Sydney, Australia (Female:11 Male:14). Tooth extraction occurred following a discussion of the possible treatment options in regard to the affected tooth. Prior to the extraction, subjects had not taken antibiotics within the previous four weeks and had no carbohydrate intake in the preceding two hours. Dentine samples were taken from the extracted teeth using sterile slow speed handpieces and sterile size one round burs. We took 4 to 5 sequential layers of 1 mm-thick dentine, starting circumferentially from the carious lesion and progressing into sound dentin (last layer). The layers ranged from layer 1, representing the most superficial zone of the lesion, to layer 5, which was the deepest part of the lesion. All sample collection was performed by a single calibrated dentist. We collected a total of 112 samples, the minimum wet weight being 7 mg/sample. Samples were suspended in 0.9% NaCl to a concentration of 1 mg in 4 µl of saline.

### pH measurement

We performed pH measurements using a minimum of 30 µl suspension of dentine samples at 1 mg per 4 µl of saline, to give a stable suspension [35]. To measure pH, we used a palladium Touch Microelectrode (Beetrode® NMPH3, World Precision Instruments Ltd., UK) with a 100 µm sensor tip. We measured pH of the samples within 15 minutes following suspension preparation. Calibration trials confirmed the stability of hydrogen ion concentration of the suspension over the limited time period of sampling. Three non-carious control teeth were also included in the pH measurement.

### DNA isolation

Purification and extraction of DNA was performed within 30 minutes from 4 µl of the prepared suspension using the QIAamp DNA Mini Kit (Qiagen, Australia) as described previously [36]–[38]. DNA concentration (*A*_260_) and purity (*A*_260_/*A*_280_) were determined using a NanoPhotometer (Implen, Munich, Germany).

### Preparation of 16S rRNA gene amplicon libraries and 454 sequencing

We used PCR to amplify microbial DNA in the dentine samples using 16S rRNA gene primers and pyrosequencing in order to examine the contents of the amplicon libraries. We targeted the phylogenetically informative V3–V4 hypervariable region of 16S rRNA gene [39], [40], using previously described primers, 331F/797R [41]. This region was targeted because it has been found to provide a higher level of bacterial coverage at the phylum and domain level, compared to other regions of the 16S gene [40], [42], [43]. These primers produced a 466 base pair long amplicon. The forward fusion primer contained sample-specific Multiplex identifier (MID) tags, which were developed by 454 Life Sciences Corp. (TCB No. 005-2009; Branford, CT, USA). We used a total of 56 MIDs (Table S1). The forward fusion primer (Roche331F) was composed of Lib-L Primer A-key, ten-nucleotide MID tag (represented by x) and 16S rRNA specific sequence (bold); 5**′**-CCATCTCATCCCTGCGTGTCTCCGACTCAG-xxxxxxxxxx- **TCCTACGGGAGGCAGCAGT**-3**′** . The reverse fusion primer (Roche797R) was composed of Lib-L Primer B-key and 16S rRNA specific sequence (bold); 5**′**-CCTATCCCCTGTGTGCCTTGGCAGTCTCAG- **GGACTACCAGGGTATCTAATCCTGTT** -3**′** . Fusion primers were assessed for hairpin, self-dimer and hetero-dimer structures by Integrated DNA Technologies, www.idtdna.com/scitools (Coralville, Iowa). We selected MIDs to provide optimum primer design.

Amplification of the V3–V4 region of the 16S rRNA gene was carried out in 25 µl reactions containing 1× FastStart High Fidelity Reaction Buffer with 1.8 mM MgCl_2_, 200 µM of each dNTP, 400 nM of each primer, 2.5 U/µl of FastStart High Fidelity Enzyme Blend (Roche Applied Science, Mannheim, Germany) and 5–250 ng of genomic DNA. Amplification was performed at 95°C for 2 min (denaturation), followed by 35 cycles of 95°C, 60°C and 72°C each for 30 s and a final elongation cycle of 72°C for 4 min. All PCR products were visually examined by electrophoresis on 2% agarose gels.

Amplicons were HPLC purified (Agencourt AMPure, Beckman Coulter, Beverly, MA, USA), randomly quality controlled (2100 BioAnalyser, Agilent Technologies, Santa Clara, CA, USA) and were sequenced unidirectionally using the GS FLX 454 Titanium platform, over two quarters of Pico Titer Plates™, at the Australian Genome Research Facility (AGRF Ltd., Brisbane, QLD, Australia).

### Filtering, de-noising, chimera check, OTU picking, alignment and taxonomic assignment of 454 sequences

The sequences from the GS FLX Titanium run were processed using the QIIME (version 1.5.0) software package [44]. Quality filtering was performed to remove sequences which were either under 360 bp or over 460 bp, contained ambiguous bases, had primer or barcode mismatches, contained homopolymers which exceeded six bases or had an average quality score below 25. The sequence length range was determined by aligning the complete dataset, prior to quality filtering, to assess the length of the majority of sequences. The remaining sequences had an average length of 429 bp. The quality-filtered sequences were de-noised [45] and chimera-checked [46] to remove sequences containing errors produced during pyrosequencing and the PCR, respectively.

Quality filtered sequences were binned into operational taxonomic units (OTUs), which are terminal nodes in a phylogenetic analysis than can be determined at different levels. We used optimal UCLUST [47] at a 97% genetic similarity which is considered to be at species level resolution [48]. Representative sequences from each OTU were aligned using PyNAST [44] against the GreenGenes core set, with a minimum length of 200 bp and identity of 80%. PyNAST aligns the short GS FLX generated sequences (360–460 bp) against the full 16S rRNA gene. We removed columns which solely contained gaps from the alignment prior to building phylogenetic trees. To overcome the difficulty in aligning highly variable 16S rRNA gene sequences, it is common to hide or lane-mask regions where at least 50% of the base composition is not conserved [49]. We did not hide variable regions because lane-masked alignments can ‘mute’ the phylogenetic diversity observed [50]. The gap-filtered sequences were taxonomically assigned using the RDP classifier and nomenclature [51]. Taxonomic classifications were also checked against the HOMD database, for which we also used the RDP nomenclature. The gap-filtered alignments were used to generate a phylogenetic tree. The phylogeny was inferred using maximum likelihood in RAxML (version 7.0.4, CIPRES webserver [52]).

### Analysis

We used linear mixed effects modelling to examine the relationship between pH and layer (fixed effects), allowing correlated slopes and intercepts to vary amongst individuals (random effects). The linear mixed effects model was fitted in R using the LME4 Package. Variation in oral microbiota among the pH groups was assessed by α-diversity (within group diversity), using both a phylogeny-based metric [53] and the total number of OTUs. As the data were sequenced at various depths, rarefaction was performed. This involved performing ten sampling repetitions without replacement, at each sequencing depth (number of sequences). Rarefaction revealed a plateauing of diversity by 1000 sequences per sample. Hence, all samples were standardised to 1000 sequences per sample, to ensure comparability between samples for all analyses.

We used linear mixed effects modelling to assess the relationship between fixed effects, pH and α-diversity (phylogenetic diversity and number of OTUs at 1000 sequences/sample), allowing correlated slopes and intercepts to vary amongst individuals (random effects). The linear mixed effects models were fitted in R using the LME4 Package.

We used Random Forest (RF) analysis to identify which species differentiate a caries associated microbial community along the pH gradient, from acidic to neutral conditions. RF was performed on the abundance of OTUs in the caries-associated microbiota under the different pH groupings. To remove bias, the samples were rarefied so that each contained 1000 sequences and only included OTUs that were observed greater than five times in the complete dataset [54]. The RF analysis was run in RF++, a RF algorithm that handles cluster correlated data (e.g. pH groups), with specification of repeated measures, in our case the layers per individual [55]. RF is a two-way comparison. As we had five pH groups, we performed a total of ten comparisons using RF analysis, to ensure all pH groups were compared to each other. We performed RF analysis using 5000 trees in the forest, and an out of bag estimate of error to determine the strength of classification of species as discriminatory. We determined that a species was discriminatory for a pH range if it had an importance score above 0.001 as in accordance with previous recommendations [54].

## Results

We isolated DNA from 110 dentine samples to determine if there is a ‘core’ microbial population associated with dentine caries and to test if the oral microbial community is influenced by pH of the lesion. The DNA samples were used to generate 16S gene (V3–V4 region) amplicons that were sequenced using 454 technology to construct genomic libraries. From two quarters of a plate (equivalent to half a plate in 454 run) in two separate runs, a total of 449,762 raw sequences were acquired by pyrosequencing that was reduced after quality filtering, de-noising and chimera checking by 14.9%, 0.8% and 19.2%, respectively. A total of 296,534 working sequences (Table S2) with an average read length of 429 base pairs were obtained. The average number of reads per sample was 3420 sequences post quality filtering. The sequence data is available in the European Nucleotide Archive (ENA), accession number PRJEB5178 (http://www.ebi.ac.uk/ena/data/view/PRJEB5178).

**Figure 1:**
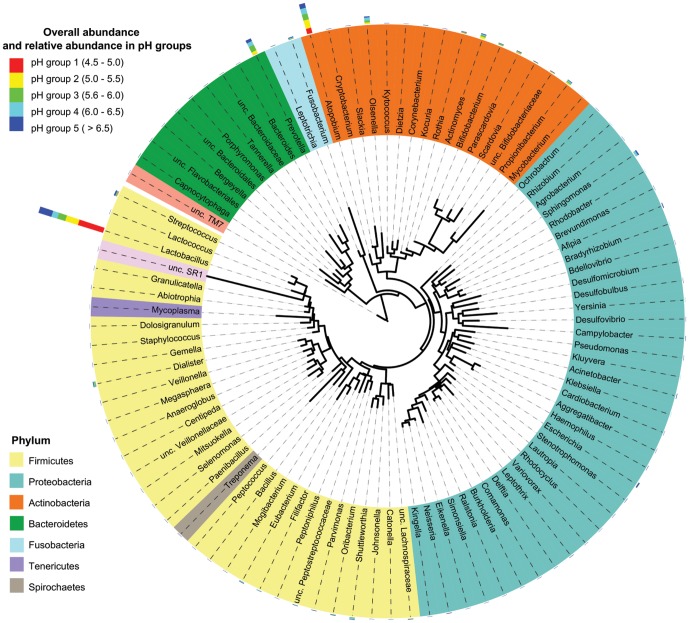
Genus level phylogenetic tree of carious lesion-associated microbiota. The phylogenetic tree was generated using maximum likelihood in the program RAxML (version 7.0.4, CIPRES webserver [52]), and graphically displayed using iTOL [71]. Genera are coloured according to their corresponding phylum. The outer band shows the overall abundance of each genus as well as relative abundance in pH group.

The sequences were clustered into species level operational taxonomic units (OTUs) at 3% genetic distance. Within the data, clustering identified the presence of 409 species (Table S3) assigned to 9 phyla and 97 genera. The dominant phyla in the dentine caries lesions were Firmicutes, Actinobacteria and Bacteroidetes, accounting for 95% of sequences (Figure 1). The phyla which accounted for the remaining 5% of sequences included Proteobacteria, Fusobacteria, Spirochaetes, TM7 and SR1 (Figure 1). The majority of sequences were classified into 12 genera including; *Lactobacillus* (40.4%), *Atopobium* (18.8%), *Prevotella* (9.4%), *Olsenella* (4.8%), *Actinomyces* (3.2%), *Streptococcus* (2.2%), *Propionibacterium* (2.1%), *Bifidobacterium* (1.2%), *Dialister* (1.7%), *Sphingomonas* (1.8%), *Fusobacterium* (1.3%) and *Parascardovia* (1.3%).

To examine whether there was a ‘core’ microbiome associated with dentine caries, the level of shared OTUs among individuals was compared. Some 50.5%+/−17.6% of OTUs were shared among the dentine caries samples from the 25 individuals investigated. As multiple (four to five) microbial DNA extracts were available per individual, it was also possible to compare the degree of inter- to intra-individual variation within the caries microbial community. We found that 65.0%+/−15.5% of OTUs were shared within an individual between the different sample layers. These results indicate a large degree of overlap in the dentine caries microbiome between individuals.

To determine variation in pH from the most superficial (layer 1) to the deepest (layer 5) zone of the lesion we used linear mixed effects modelling (Table S4). This analysis revealed a highly significant relationship between the lesion depth and pH. The most superficial zone was found to be significantly more acidic than the deepest areas of the lesion sampled (p<0.0001, Table S4a).

**Figure 2:**
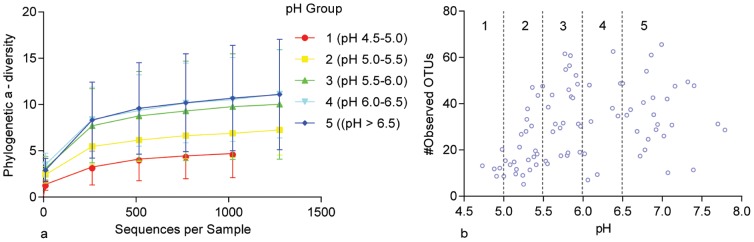
α-diversity with reference to pH. The α-diversity between dentine caries samples from acidic to neutral conditions from phylogenetic analysis (a) and the number of OTUs (b). The number of OTUs was determined from samples that contained 1000 sequences and only included OTUs which occurred above five times.

To test whether the microbial community composition associated with dentine caries was influenced by the changing pH within the lesion, we compared the degree of shared sequences within groups, also termed *α*-diversity, by both a phylogenetic diversity metric and the overall number OTUs. Linear mixed effects modelling of *α*-diversity (1000 sequences/sample) and pH revealed that bacterial communities from carious lesions under more acidic compared to more neutral conditions had severely reduced phylogenetic diversity (p<0.0001 Table S4b, Figure 2a) and less numbers of different OTUs (p = 0.0319 Table S4c, Figure 2b).

**Figure 3:**
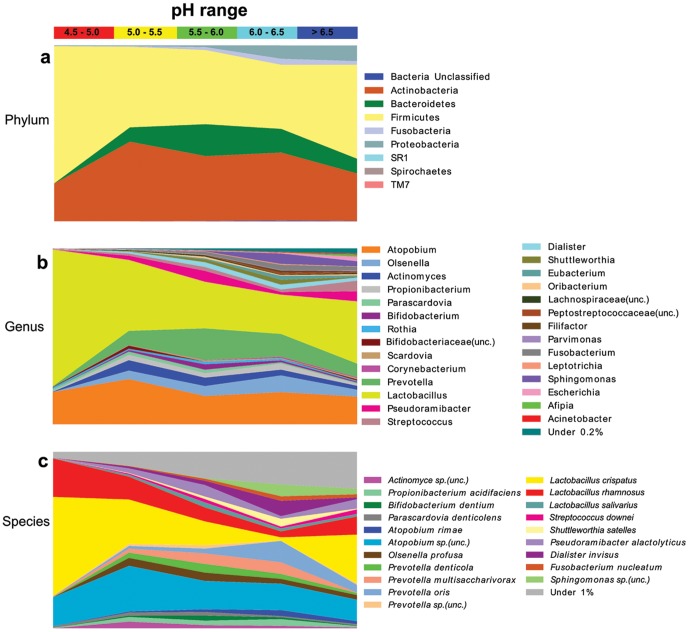
pH gradient and relative abundance at phylum, genus and species level. Relative abundance of phyla (a) genera (b) and species (c) across the pH gradient in carious lesions was calculated from samples that each contained 1000 sequences and only included OTUs which occurred above five times. For full list of species level taxa see Table S3.

The overall disparity in diversity observed between microbial communities under acidic and neutral conditions was associated with changes at both the phylum and genus level (Figure 3). For all abundance comparisons, samples were standardised to contain 1000 sequences per sample. While Firmicutes were the most dominant phylum across all pH groups, they were present in higher proportions in the most acidic conditions (78%, pH 4.5–5.0), compared to less acidic conditions (36–53%, pH 5.0–>6.5). In communities from the most acidic lesion samples, the Firmicutes phylum was primarily represented by the *Lactobacillus* (77%) genus. In comparison, microbial populations from zones above pH 5.5 had lower frequencies of *Lactobacillus* (22–40%), and greater amounts of *Streptococcus*, *Pseudoramibacter* and *Dialister*. As the proportion of Firmicutes in the caries microbiome decreased with increasing pH, other phyla became more dominant, including Bacteroidetes, Fusobacteria and Proteobacteria (Figure 3a). Despite the low abundance of Proteobacteria within all pH groups, numerous genera within this phylum were detected at more neutral pH ranges (>6.0, 10.6%), creating high heterogeneity of genera detected in caries (Figure 3b). Across the pH gradient the proportion of Actinobacteria remained relatively constant (21–45%). Within the Actinobacteria phylum, the dominant genus observed in the caries dentine samples was *Atopobium*. The change in relative abundance of bacterial species along the pH gradient is shown in Figure 3c.

Random Forest (RF) analysis was used to identify which species differentiate a dentine caries community that is under acidic or neutral conditions. RF is a classifier that recognises patterns, non-linear relationships and dependencies between data, in our case OTUs, within complex datasets [56]. The goal of RF is to classify the input data, given the known groupings, assess the strength of this classification and identify features which are important to the classification [56]. As the grouping classification (pH range) was known, this method was used to identify the OTUs that best discriminated microbial communities at different pH ranges. This analysis also identified those species that did not discriminate between pH groups, and hence could be identified as common/core species present in dentine caries (Table S5).

**Figure 4:**
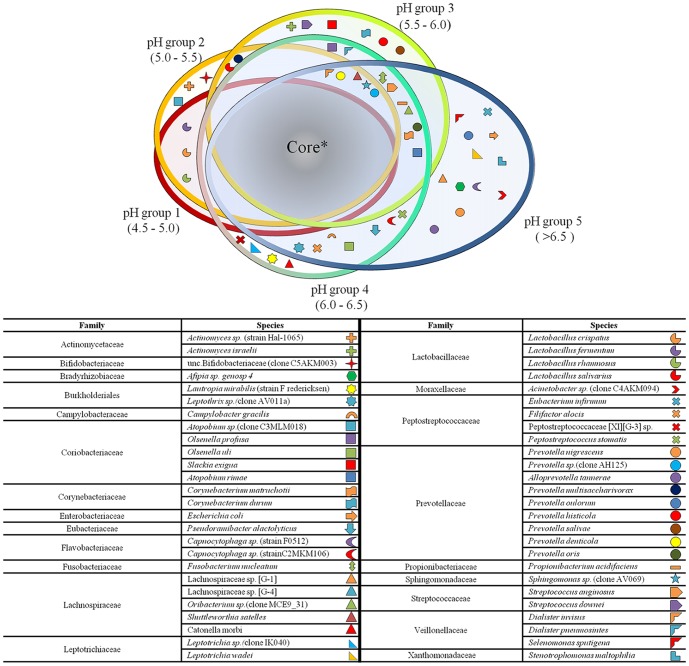
Substantial core model. The pH-driven ‘substantial core model’ describes the influence of pH on the microbial population of carious lesions. The model was constructed from Random Forest analysis results (Table S5 and S6). Bacteria presented in the figure are discriminatory of pH values. Along the pH gradient, fewer bacterial species were distinctive of acidic pH ranges than neutral pH values (Table S5). We found that 58% of dentine caries associated bacterial taxa were omnipresent and were not discriminated by pH. These comprise the core* part of this model (Table S6).

RF revealed that 2 species were highly predictive of communities associated with the most acidic conditions (pH group 1, 4.5–5.0), and 20 species were highly predictive of neutral (pH group 5, >6.5) pH-associated communities (error rate, 16.1%, Table S5). Anchor species for the most acidic pH range included *L. fermentum* (Importance Score = 0.0401) and *L. rhamnosus* (Importance Score = 0.013). A diverse range of anchor species were specific for the highest pH group (>6.5), including *Sphingomonas sp.* (Importance Score = 0.0476), *Lachnospiraceae sp.* (Importance Score = 0.0115) and *Streptococcus oralis* (Importance Score = 0.0062). There were also predictive species for communities associated with a mid-range pH (5.0–6.0) compared to neutral pH conditions. We found that 9 species were predictive for communities associated with a pH range of 5.0–5.5 (error rate, 20%, Table S5), dominated by *Prevotella* and *Actinomyces* species. As the pH became less acidic (pH range of 5.5–6.0) 5 predictive species (error rate, 48.1%, Table S5) were identified. These included *Shuttleworthia satelles* (Importance Score = 0.0016), *Prevotella multisaccharivorax* (Importance Score = 0.0105) and *Propionibacterium acidifaciens* (Importance Score = 0.0024). RF analysis also revealed that 58% of the caries-associated taxa were not distinctive of any investigated pH range. The microbiota which appeared to be unaffected by pH included species of *Leptotrichia* and *Prevotella*, in addition to *Streptococcus salivarius* and candidate division *TM7 [G-1] sp.* (see Table S6). The accumulated results from RF analysis are presented in the proposed ‘substantial core model’ in Figure 4.

## Discussion

The composition of dentine caries-associated microbiota was observed to shift with pH gradient, supporting the postulate of the extended ecological plaque hypothesis. The finding of a core population of bacteria, unaffected by pH, has enabled us to propose a ‘substantial core model’ for dentine caries. By detailed investigation, we were able to draw broad conclusions regarding variation in the oral bacterial community between cavitated lesions and biofilms associated with health and caries. Further, by linking data to an ecological variable, pH, it was possible to infer the relationship between dentine caries, pH and the microbiota.

While technically, the inclusion of plaque bacterial population and plaque pH from healthy subjects may provide a control, we found this comparison of little biological relevance for the question of carious dentine and pH. In other words, we only present the bacterial population in the very late stages of caries and its relevance to the pH of the lesion. In plaque bacterial studies, the bacterial population can be compared in active or inactive disease states or between caries-free and caries-active individuals [31], [57]–[59]. However, unlike plaque studies, there is no equivalent biologic control available for an established lesion where the bacterial DNA is directly extracted from the caries mass. Hence, we used published literature on health and caries to compare broadly to our dentine caries study.

### Caries-associated microbiota is lower in diversity than health-associated oral microbiota

Comparison of the bacterial profile in the present study to data available for oral biofilms associated with health indicates substantial differences in community diversity regardless of pH of the lesion, which is not yet addressed in current literature. We found that caries- compared to health-associated microbiota is lower in diversity and varied in composition at both the broad (phylum) and detailed (genus) level. The carious lesions we investigated contained between 15% to 50% of the total number of OTUs recorded from pyrosequencing analysis of plaque and saliva samples from healthy sites [60] and healthy mucosal biofilms [58], respectively. While Firmicutes, Actinobacteria and Bacteroidetes accounted for 95% of sequences in our investigated carious lesions, these phyla comprised only 68% of sequences from healthy sites [58]. A major disparity at the phylum level related to Proteobacteria which accounted for 15–22% of bacteria in healthy sites [58], [60], but only 4.4% of the sequences from carious lesions. We observed disparity between some of the most abundant genera in health [58], [60] compared to our dentine caries study including: *Streptococcus*, *Neisseiria*, *Corynebacterium*, *Rothia*, *Haemophilus* and *Veillonella*. While the greater microbial diversity in healthy sites compared to carious lesions was related to higher levels of the above outlined genera, we did find a selection of genera that were exclusively abundant in dentine caries: *Lactobacillus*, *Atopobium*, *Olsenella*, *Propionibacterium*, *Bifidobacterium*, *Dialister*, *Sphingomonas* and *Parascardovia*.

### The microbial population in cavitated lesions of dentine is different from non-cavitated caries samples

Previous investigations of caries-associated microbiota have primarily analysed saliva [31] or plaque [33]. While the oral microbiota is a continuum, community composition is known to vary at different intra-oral sites due to varying ecological conditions [61]. We found that caries-associated microbial populations from the active site of decay differed from non-active site samples [31], [33] being lower in diversity and varied in community structure. For instance, pyrosequencing analysis of saliva microbiota from caries-active individuals revealed highly diverse communities, comprising 600 to 4200 species-level phylotypes in each sample, with great inter-individual variation [31]. We found a much less diverse microbial community within cavitated carious lesions, with 5 to 66 phylotypes recorded per sample, and a high representation of shared species (50.5%+/−17.6%) between individuals. Our findings more closely match results from pyrosequencing analysis of plaque samples adjacent to the margin of the cavity that contained between 73–120 OTUs per sample [33]. The diversity difference observed in the carious lesion compared to non-active site samples indicates that the site of sampling influences the interpretation of the relationship between microbiota and disease.

The variation in reported microbiota diversity between the present study and others due to sample type, impacts significantly on the interpretation of which taxa are associated with disease. As an example, *Prevotella* was the only over-abundant (25%) genus reported in saliva samples from caries-active individuals [31]. In the present study, the relative abundance of *Prevotella* at genus level was not distinctive from that found in healthy dental biofilms (carious = 9.4%, healthy = 4.1–19.7%) [31], [58], [60]. However, Random Forest analysis revealed that members of the *Prevotella* genus including *Prevotella* sp. (strain B31FD), *P. histicola* and *P. multisaccharivorax* were distinctive of more acidic pH ranges in carious dentine, while other *Prevotella* species, such as *P. buccae*, *P. dentalis* and *P. nigrescens*, were shared among the core microbiota (Table S6). The above-mentioned study also found *P. histicola* distinctive of saliva in caries-active individuals [31]. Thus, sample type and species or strain level resolution, in addition to a key ecological variable such as pH, is required in caries studies to better understand disease progression.

### The presence of a pH-driven substantial core

Alkali production, acid-utilization and acid production by bacteria is influenced by environmental pH [28], [62], with more acidic environments favouring development of caries. Previous research examining the relationship between caries, microbiota and pH, has focused on the use of chemostat-controlled conditions [30], as well as different buffered and un-buffered pH values [28]. DNA- and RNA-based Special Isotope Probing (SIP) demonstrated the metabolic activity of cloned bacterial species at different pH values. In supragingival plaque samples of caries-free children, members of the genera *Streptococus*, *Neisseria*, *Veillonella* and *Granulicatella* were capable of metabolizing isotopically labelled glucose under acidic (pH 5.5) and neutral (pH 7) conditions [28]. We detected these genera at relatively low numbers and some were anchor species for less acidic to neutral pH zones (e.g. *Streptococcus anginosus*, *Streptococcus oralis* and *Dialister invisus*), suggesting that these genera are obtruded in the course of caries progression. Other taxa, including both proteolytic and saccharolytic *Prevotella* sp., have been found to be capable of metabolic activity in mildly acidic conditions [63], [64]. We found *Prevotella* taxa were anchor species for the mid-range pH value (5.5–6.0). Our work has demonstrated a wider range of bacteria characteristic of mildly acidic to neutral conditions than detailed above. These include *Prevotella oulorum*, *Alloprevotella tannerae* (formerly known as *P. tannerae* [65]), *Leptothrix* sp. (clones CA004 and AV011a), *Eubacterium infirmum*, *Rothia dentocariosa*, *Actinomyces israelii*, *Slackia exigua* and *Peptostreptococcus stomatis*.

A recent report based on *in vitro* findings showed that extended incubation at pH 4.5 markedly reduced bacterial diversity of health-associated oral microbiota [28]. This accords with the decline of diversity at low pH observed in our study (Figure 2). *Lactobacillus* species including *L. fermentum*, *L. rhamnosus* together with a *Propionibacterium* species, were abundant at controlled pH 4.5 [28]. We also found *L. rhamnosus and L. fermentum*, to be distinctive species at low pH ranges (4.5–5.5) in carious lesions. Further, we detected low levels of *Streptococcus mutans*, a well-studied caries-associated species, in dentinal lesions [20]. Nonetheless, *Streptococcus downei*, which until 1988 was believed to be *S. mutans* Serotype h [66], was detected as one of the low pH-associated species. The present study in particular demonstrates the paucity of *S. mutans* and *S. sobrinus* in advanced lesions. The role of these bacteria in caries *initiation* is well presented in the literature [10], [12], [17], [67]–[69]. However, these species are known to be scarce in advanced carious lesions [25], [36], [38]. Further, more recent sophisticated methods assigns little role for these bacteria even in caries initiation [20], [70].

We also found a substantial proportion (58%) of dentine caries-associated taxa were present along the pH gradient from 4.5–7. The metabolic activity or contribution of these dentine caries-associated microbiota, which make up the core with reference to pH cannot be determined directly from our taxonomic data. However, the presence of these organisms along the pH gradient may reflect the ability of core species to be metabolically active under a range of pH conditions.

By determining which microbial species differentiate a dentine caries community that is under acidic or neutral conditions, the present study provides insight regarding disease progression in carious lesions. The microbiota associated with the most acidic pH ranges, including *L. fermentum* and *L. rhamnosus*, potentially represent taxa that are important for progression of the lesion. In contrast, the microbiota associated with the more basic pH ranges, such as *Sphingomonas sp*, *S. oralis*, *Lachnospiraceae sp.*, *Atopobium rimae* and *Bifidobacterium dentium*, are potentially health-associated microbiota. Those taxa distinctive of low pH represent potentially important bacteria in disease progression from initial to more advanced caries including infection of the pulp. Hence, these are potential targets for anti-microbial strategies to arrest progression of caries.

### Summary

The carious process is a continuum of events and this study presents a portion of this continuum from a novel perspective. Consortium behaviour within a bacterial community depends on environmental variables that have been overlooked in previous studies. The pH of the environment can be a result of acidogenicity of bacteria combined with or without imposed pH from external sources, such as food and beverages. Nonetheless, it is believed that a constant interaction between the two exists [26] in which the substrate availability can further direct and impose a shift in bacterial population and a sustained ecology, leading to caries progression. As stated earlier, we propose that without taking into account a defining ecological variable, such as pH of the environment, a cross-sectional bacterial analysis of caries microbiota, although still informative, remains deficient to assign a key role for specific bacteria, unless a longitudinal analysis of bacteria is carried out at different stages of the caries process. We addressed this issue by comprehensively cataloguing bacteria associated with carious lesions and considering the influence of pH, on microbial composition. Our proposed ‘substantial core model’ identifies pH-distinctive taxa and demonstrates bacterial diversity changes from acidic to neutral pH gradients. The presence of numerous species that are not discriminatory of pH and are omnipresent in this ecology represents the core of the bacterial consortium in dentine caries with respect to pH. The present in-depth report of bacterial community constitution affirms the ecological plaque hypothesis at the *in vivo* level.

## Supporting Information

Table S1 Details of the sample IDs, oligonucleotide description and MIDs and pH in Pool 1 and 2.
(XLS)

Table S2 Details of the processed sequences from raw sequences to quality filtering, de-noising and chimera check.
(XLS)

Table S3 Clustered sequences into species level Operational Taxonomic Units at 97% similarity with HOMD.
(XLS)

Table S4 Linear Mixed Effects Models.
(XLS)

Table S5 Results from Random Forests classifier of 97% ID OTUs (species-level phylotypes) that discriminate the pH groups.
(XLS)

Table S6 The bacterial taxa (HOMD and rdp format, S6a and b) which are discrimintatory of different pH values, and the taxa which form the ‘core’ that are non-discriminatatory for pH. These results are obtained from the combined Random Forest analysis, comparing the pH groups.
(XLS)
